# Electrophysiological Assessment of Injury to the Infra-patellar Branch(es) of the Saphenous Nerve during Anterior Cruciate Ligament Reconstruction Using Medial Hamstring Auto-grafts: Vertical versus Oblique Harvest Site Incisions

**DOI:** 10.5812/atr.11146

**Published:** 2013-12-01

**Authors:** Reza Tavakoli Darestani, Mohammad Mehdi Bagherian Lemraski, Mehrdad Hosseinpour, Amin Kamrani-Rad

**Affiliations:** 1 Orthopedics Surgery Department, Beheshti University of Medical Sciences , Tehran , IR Iran; 2 Trauma Research Center, Kashan University of Medical Sciences, Kashan, IR Iran

**Keywords:** Arthroscopy, Anterior Cruciate Ligament Reconstruction, Medial Hamstring Tendons, Infrapatellar Branch of the Saphenous Nerve

## Abstract

**Background:**

It was suggested that the direction of incision for medial hamstring tendons harvesting influences the incidence of injury to the infrapatellar branch of the saphenous nerve (IPBSN), a common complication following arthroscopically-assisted anterior cruciate ligament reconstruction (ACLR).

**Objectives:**

The main purpose of current study was to compare the incidence of IPBSN injury between vertical and oblique incisions utilizing electrophysiological evaluation.

**Patients and Methods:**

There were 60 patients underwent arthroscopically-assisted ACLR assigned to two equal vertical or oblique incision groups, randomly. One year postoperatively, the patients were electrophysiologically examined to detect whether IPBSN is injured. The Lysholm score was completed. The patients' satisfaction with surgical outcomes determined utilizing visual analogue scale (VAS). Finally, two groups were compared and the effect of IPBSN injury on function and satisfaction was investigated.

**Results:**

The incidence of IPBSN injury was higher in the vertical group (4 patients vs. 10 patients), but the difference was not statistically significant. The mean of Lysholm and VAS scores were the same. Also, the mean of Lysholm score was the same in patients with and without IPBSN injury. However, patients without IPBSN injury were more satisfied (8.9 ± 9 vs. 7.4 ± 1.1; P < 0.001).

**Conclusions:**

IPBSN injury is a common complication following arthroscopically-assisted ACLR and, if not significant, oblique direction of the incision is associated with decreased incidence of the injury. IPBSN injury has no effect on the function but because of the disturbance with patients' satisfaction, authors believe the oblique incision is preferable to avoid the nerve injury during medial hamstring tendons harvesting.

## 1. Background

Arthroscopically assisted ACL reconstruction (ACLR) technique is one of the most commonly used surgical procedures recreating the normal kinematics of the knee and ligamentous stability (1-6). Recent studies have shown the benefits of auto-grafts from semitendinosus and gracilis tendons as a safe and effective ACL graft (1, 7). However, arthroscopically-assisted ACLR using medial hamstring tendons is not free from complications (3, 4, 6, 8). One of the complications associated with the incising on the medial aspect of the proximal tibia to harvest the medial hamstring tendons is altered sensation in the upper medial part of the lower leg (1, 3, 8-11). The purelysensory infrapatellar branch (es) of the saphenous nerve (IPBSN) supplies the skin over the medial and front aspect of the knee and passes with an oblique traction the incision site through which medial hamstring tendons are harvested (1, 4, 10). This anatomical location exposes the IPBSN to the potential risk of injury resulted from the incision (6, 9). The incidence of iatrogenic injury to IPBSN after knee arthroscopy has been reported 12% to 84% (2, 9, 12, 13). Injury to IPBSN may result in dysesthesia, hypoesthesia, neuroma, reflex sympathetic dystrophy, anterior knee pain and kneeling pain interfering with patients’ gratification (5, 9, 13-18). Also it is reported that loss of sensation is a source of concern for patients and affects their activities (2 - 12%) (2, 13, 16). Some authors suggested that based on the anatomical findings of the IPBSN distribution, the orientation of the incision for harvesting the graft, theoretically, can influence the risk of IPBSN damage (1, 3, 4). However there is no consensus on the most appropriate incision direction of pesanserinus area for hamstring tendon harvesting and the results are controversial (2, 10, 17). In addition, the studies compared the incidence of IPBSN injury in different incision directions used physical examination and questionnaires and to our knowledge there is no electrophysiological comparison as a reliable method.

## 2. Objectives

The main purpose of current study was to electrophisiologically compare the incidence of injury to IPBSN after arthroscopically ACLR with vertical and oblique incisions to harvest medial hamstring tendons. It was hypothesized that oblique incision can reduce the incidence of IPBSN injury.

## 3. Patients and Methods

**Figure 1.**
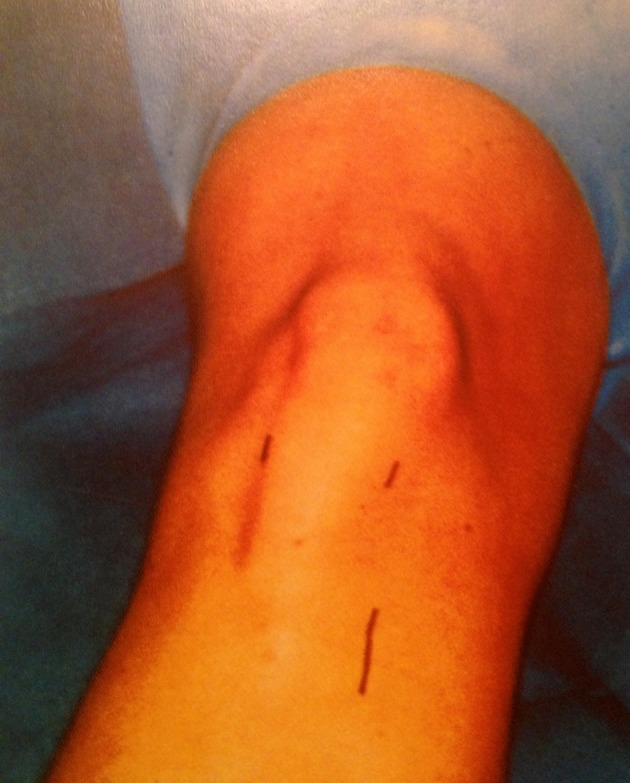
Vertical Incision

**Figure 2.**
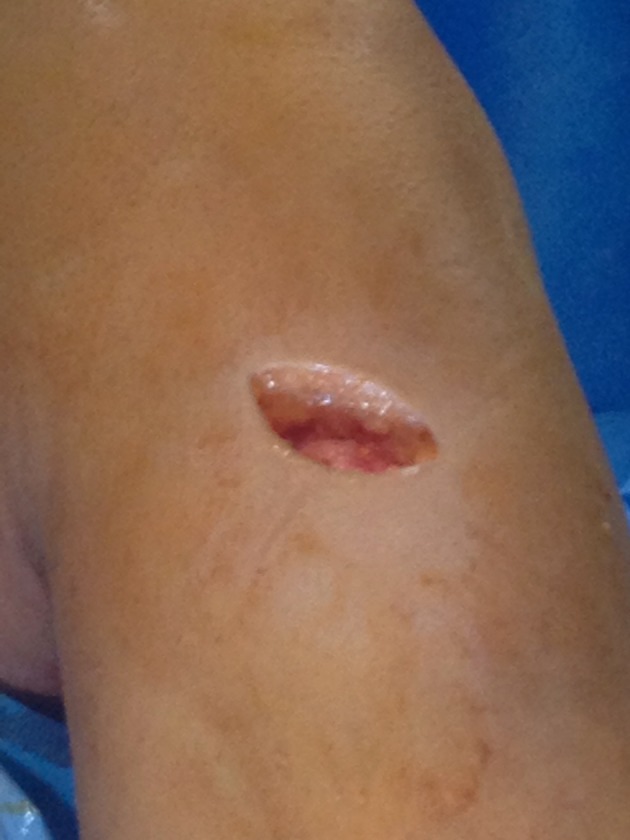
Oblique Incision

We have prospectively studied 60 patients with complete ACL tear with or without meniscus injury that underwent arthroscopic-assisted ACLR using auto-graft from medial hamstring tendons between 2006 and 2009. The study was approved by Institutional Review Board and, before surgery; informed consent was obtained from patients. The patients were randomly assigned to two equal groups: vertical (Figure 1) and oblique incisions (Figure 2) over the pes anserinus region. The incisions in two groups sized 3 cm. Medial hamstring tendons were retracted using a tendon hook and harvested with a tendon stripper. The groups were matched with age, sex, and BMI (Table 1). Patients with a history of any previous leg surgery or neurologic deficit, and who had any associated ligamentous injury which required surgical management, were excluded. All patients were operated on by the same surgeon (R. T. D) and same technique (endo-button). 

**Table 1.** Comparison of the Demographic Characteristics of Two Groups

	Vertical N = 30	Oblique N = 30	P Value
**Age, Mean ± SD, y**	28.5 ± 6.5	29.6 ± 6.1	N.S ^a^
**Gender**			N.S ^a^
Male	21	23	
Female	9	7	
**BMI, Mean ± SD, kg/m**	24.9 ± 2.9	23.7 ± 2.9	N.S ^a^
**Meniscal Injury**	14	11	N.S ^a^

^a^ No significance.

All the patients underwent identical postoperative rehabilitation protocols. Immediate range of motion exercises was begun and patients wore a functional knee brace in full extension for first postoperative week, which was then adjusted to allow 90˚ of flexion for another 3 weeks. Full weight bearing was allowed when tolerated. One year after surgery patients were assessed to determine the incidence of injury of IPBSN in each group. Simultaneously, the skin on both lower legs was touched lightly and patients were asked to demonstrate any altered sensation felt in the operated leg by comparing to the non-operated side. The injury to IPBSN was considered clinically if patient felt hyposthesia at the anterolateral aspect of the knee and proximal tibia. An electrophysiological study of the nerve conduction was done based on the method described by Bademkiran et al. (19). Electric stimulation was applied through a surface electrode placed medially on the skin 2 cm below the patella and sensory nerve action potentials were recorded using a needle electrode inserted close to the nerve 1 cm lateral to the femoral artery in the inguinal region. Finally, all patients completed the Lysholm score to investigate if altered sensation after ACLR affected their function. Also, the patients were asked to mark their satisfaction with the outcomes of the surgery using visual analogue scale (VAS). In this scale, 0 considered as no and 10 as maximal satisfaction. The incidence of clinical and electrophysiological IPBSN injury was compared between groups using Fisher’s exact test. Satisfaction (VAS) and Lysholm score were compared between two groups and between patients with and without IPBSN injury using independent samples t-test. SPSS statistical software (version 15.0; SPSS, Chicago, IL) was utilized to perform Statistical analysis. P value < 0.05 was considered significant.

## 4. Results

Table 2 shows the outcomes of the study compared between the two groups. Electrophysiological studies revealed that the incidence of IPBSN injury was higher in the vertical group (4 patients vs. 10 patients), but the difference was not statistically significant. Interestingly, there was complete correlation between electrophysiological and clinical examinations (touching) to detect the nerve injury. The mean of Lysholm and VAS scores were the same in the vertical and oblique groups. Also, the nerve injury did not affect the Lysholm score (89.9 ± 9.8 in patients without IPBSN injury versus 91.9 ± 8.5 in patients with IPBSN injury), but we found that patients without IPBSN injury were more satisfied than patients in whom IPBSN was injured (8.9 ± 9 in patients without IPBSN injury versus 7.4 ± 1.1 in patients with IPBSN injury; P < 0.001). 

**Table 2.** Comparison of the IPBSN Iatrogenic Injury, Lysholm Score and Satisfaction Between Vertical and Oblique Incision Groups

	Vertical N = 30	Oblique N = 30	P Value
**IPBSN injury**	10	4	N.S
**Lysholm score**	89.5 ± 10.7	91.1 ± 8.1	N.S
**Satisfaction (VAS)**	8.6 ± 1	8.5 ± 1.3	N.S

## 5. Discussion

This study demonstrated that the direction (oblique or vertical) of the incision over the pes anserinus region through which the medial hamstring tendons are harvested, has no significant effect on the incidence of IPBSN injury. However, if not significant, vertical incision was associated with higher incidence of IPBSN injury and it seems that if there had been more patients contributed in current study, the difference would have been significant. It is well documented that IPBSN is likely to be damaged during ACLR (3, 5, 9, 10, 14). However, there are a few studies addressed this complication; most of them refer to bone- patellar tendon- bone technique. It is that the incidence of the IPBSN injury after ACL reconstruction using medial hamstring grafts is unclear (2, 3, 5, 9). Cadaveric studies revealed that when the saphenous nerve leaves the adductor canal divides into two terminal branches: the sartorial branch and the infrapatellar branch which runs in the anterior region of the knee in a slightly oblique manner and supplies the skin over the anteromedial aspect of the knee between patellar apex and tibial tuberosity in 98.5% of cases (16, 20). The incision, through which medial hamstring tendons are harvested, is made over the insertion at the pes anserinus, with 2.5 - 4 cm distance from the tibial tuberosity (1, 2). In this region, harvesting the tendons from their insertion in pes anserinus can damage the IPBSN (9, 10, 16, 17) resulting in hyposthesia of the anteromedial aspect of the knee (9).

The incidence of iatrogenic injury to IPBSN after knee arthroscopy has been reported 12% to 84% (2, 9, 12, 13) with a hyposthetic area measured as 25 to 53.2 cm^2^ (2, 16, 21). The high incidence and potential adverse effects of the IPBSN injuries during arthroscopic ACLR had propelled some authors to develop techniques such as newer graft-harvest strategies, to minimize the injury and subsequent sensory changes (10, 22, 23). Muchizuki et al. found that at least one branch of the medial femoral cutaneous nerve and the saphenous nerve in infrapatellar region crosses the longitudinal incision through which the medial hamstring tendons are harvested in 88% of the cases. They demonstrated that these nerve branches exist on the insertion of the sartorius muscle between gracilis and semitendinosus tendons and incising the skin in an oblique manner can effectively minimize the injury to the sartorius insertion and subsequent sensory disturbance (4).

Based on the findings by Muchizuki et al. and others who suggested that changing the direction of incision decreases the incidence of IPBSN injury during ACLR (3, 4, 24), we hypothesized that the oblique incision reduces the rate of the IPBSN injury. To our knowledge this is the first study in which the incidence of IPBSN injury is compared between vertical and oblique incision directions electrophysiologically. Our findings did not confirm the hypothesis and NCV showed the incidence of denervation is statistically the same between two groups. However, as mentioned before one should consider that there were more patients with IPBSN injury in vertical group and we assume that if there had been more patients in current study, we would have found a significant difference between two groups which needs to be investigated in future studies.

Furthermore, as Figueroa et al. demonstrated (9), we found that the electrophysiological findings are completely in accordance with clinical findings. Although, the studies in which the incidence of injury to IPBSN is assessed and/or compared between different incision directions have reported different rates of the injury, but to our knowledge there is no study that supports the benefits of vertical incision over the oblique or horizontal incisions (Table 3). Like our finding, Kjeagaard et al. reported that the incidence of the injury to the IPBSN and the hyposthetic area were the same between vertical and oblique groups (2). However, they found an incidence of 88% of injury to IPBSN in each group while in current study the incidence of nerve injury was higher in vertical group. Others reported that the incidence of the injury and hyposthetic area, if measured, is significantly lower in oblique or horizontal incisions compared with vertical incision in ACLR using medial hamstring tendons (3, 10) or patellar tendon (5). 

**Table 3.** Summary of Studies Assessed the IPBSN Injury Following Incising the Skin Over the Pes Ancerinus Region During ACLR Based on the Incision Direction

Authors	Incision Direction	No. of cases	Type of Autograft	Incidence of	Comment
**Muchizuki et al. (16)**	Vertical	86	Hmastring	55%	Sensory change was frequently found with a vertical incision. Daily living was only slightly affected
**Portland et al. (5)**	Vertical vs. horizontal	42 (horizontal) vs. 34 (vertical)	BPB	59% (vertical) vs. 43% (horizontal)	Horizontal incision may be a useful option to provide a more satisfactory scar with less risk of IPBSN damage
**Papastergiou et al. (**10**)**	Vertical vs. horizontal	116 knees (Vertical) vs. 114 Knees (Horizontal)	hamstring	39.7% (vertical) vs. 14.7% (horizontal)	The horizontal incision was associated with less chance of IPBSN
**Lou et al. (3)**	Vertical vs. Oblique	35 (vertical) vs. 25 (Oblique)	hamstring	65.7% (Vertical) V.s 24% (Oblique)	Oblique incision with less risk of damage for IPBSN may be better for graft harvesting
**Sanders et al. (11)**	Vertical	164	hamstring	32% (Concomitant injury of SBSN and IPBSN). 19% Isolated injury of IPBSN	No comment
**Figueroa et al. (16)**	Vertical	21 (22 knees)	hamstring	68%	The sensory loss does not impair normal daily activities
**Kjeagaard et al. (2)**	Vertical vs. Oblique	25 (Vertical) vs. 25 (Oblique)	hamstring	84% in each group	Incision direction did not affect the incidence of IPBSN Injury

The different results in studies may be resulted from different sizes of incision and different examination techniques. Some authors used standard incision sizes; however, others especially in retrospective studies assessed patients with different skin incision sizes. In addition, some authors utilized questionnaires to investigate the presence of sensory changes and measure the affected area. To date, only Figueroa et al. used electrophysiological examination as a standard evaluation method. However, Figueroa et al. found no relation between size of incision or distance from the tibial tuberosity and the presence of sensory disturbance and concluded IPBSN is injured during tendon harvesting and not during incising the skin (9). Furthermore, others have suggested that nerve injury may be occurred during skin incision, subcutaneous dissecting, medial hamstring harvesting and portal placement (4, 11, 16, 17, 25). Some authors tried to define a safe zone to harvest the medial hamstring tendons and decrease the incidence of nerve injuries. Boon et al. determined distinct safe areas and incision angles for right (3.7 - 5.5 cm distance from tibial tuberosity and 51.6°) and left (3.6 - 4.9 cm distance from tibial tuberosity and 52.5°) knees and suggested that using these results may help surgeons to avoid cutaneous nerves injuries (1). Also Ebrahein and Mekhail introduced a safety zone to avoid injury of the IPBSN (25). In spite of these findings, Muchizuki et al. could not found a safe zone to harvest the medial hamstring tendons and demonstrated that the complicated anatomic variation of the nerve branches and the overlapping distribution territories of the saphenous nerve and the medial femoral cutaneous nerve prevent determining a safe zone. However they suggested that an oblique incision is more likely to avoid nerve injury in infra-patellar region (4).

Although, there was no functional difference between patients with and without IPBSN injury, but the current study showed that sensory disturbance in patients with IPBSN injury results in lower satisfaction with the outcomes which remind us the need for techniques to decrease the incidence of cutaneous nerve injuries during incising the skin over the pes anserinus region. Kjeagaard et al. showed there is no difference in the Lysholm score between patients with and without IPBSN injury (2). Muchizuki et al. observed that IPBSN injury occurred in 43% of the patients after ACLR using medial hamstring tendons through a vertical incision and the nerve injury had significantly affected the activities of daily living (ADL) only in 3.8% (1 patient) of these patients. Also, there were 3 patients (11.5%) who experienced slight limitation in ADL due to the nerve injury (16). Although there were 30 patients in each group, but authors believe that the present study was limited by small number of the patients which would affect the statistical results. Also, we did not measure and compare the hyposthetic area because of the lack of a validated measurement method.

Based on the findings of current study, IPBSN injury is a common complication following arthroscopically assisted ACLR and, if not significant, oblique direction of the incision is associated with decreased incidence of the injury. IPBSN injury has no effect on the function, however, it is necessary to reduce the incidence of the IPBSN injury which results in dissatisfaction with the treatment outcomes. It is that the authors believe the oblique incision is superior to vertical incision to avoid the nerve injury during medial hamstring tendons harvesting, which is in accordance with the findings of previous studies (4, 9, 10).

## References

[1] Boon JM, Van Wyk MJ, Jordaan D (2004). A safe area and angle for harvesting autogenous tendons for anterior
cruciate ligament reconstruction.. Surg Radiol Anat..

[2] Kjaergaard J, Fauno LZ, Fauno P (2008). Sensibility loss after ACL reconstruction with hamstring
graft.. Int J Sports Med..

[3] Luo H, Yu JK, Ao YF, Yu CL, Peng LB, Lin CY (2007). Relationship between different skin incisions and the injury of the
infrapatellar branch of the saphenous nerve during anterior cruciate ligament
reconstruction.. Chin Med J (Engl)..

[4] Mochizuki T, Akita K, Muneta T, Sato T (2003). Anatomical bases for minimizing sensory disturbance after
arthroscopically-assisted anterior cruciate ligament reconstruction using medial
hamstring tendons.. Surg Radiol Anat..

[5] Portland GH, Martin D, Keene G, Menz T (2005). Injury to the infrapatellar branch of the saphenous nerve in anterior
cruciate ligament reconstruction: comparison of horizontal versus vertical harvest site
incisions.. Arthroscopy..

[6] Soon M, Neo CP, Mitra AK, Tay BK (2004). Morbidity following anterior cruciate ligament reconstruction using
hamstring autograft.. Ann Acad Med Singapore..

[7] Aglietti P, Buzzi R, Menchetti PM, Giron F (1996). Arthroscopically assisted semitendinosus and gracilis tendon graft in
reconstruction for acute anterior cruciate ligament injuries in
athletes.. Am J Sports Med..

[8] Kodkani PS, Govekar DP, Patankar HS (2004). A new technique of graft harvest for anterior cruciate ligament
reconstruction with quadruple semitendinosus tendon autograft.. Arthroscopy..

[9] Figueroa D, Calvo R, Vaisman A, Campero M, Moraga C (2008). Injury to the infrapatellar branch of the saphenous nerve in ACL
reconstruction with the hamstrings technique: clinical and electrophysiological
study.. Knee..

[10] Papastergiou SG, Voulgaropoulos H, Mikalef P, Ziogas E, Pappis G, Giannakopoulos I (2006). Injuries to the infrapatellar branch(es) of the saphenous nerve in anterior
cruciate ligament reconstruction with four-strand hamstring tendon autograft: vertical
versus horizontal incision for harvest.. Knee Surg Sports Traumatol Arthrosc..

[11] Sanders B, Rolf R, McClelland W, Xerogeanes J (2007). Prevalence of saphenous nerve injury after autogenous hamstring harvest: an
anatomic and clinical study of sartorial branch injury.. Arthroscopy..

[12] Jameson S, Emmerson K (2007). Altered sensation over the lower leg following hamstring graft anterior
cruciate ligament reconstruction with transverse femoral fixation.. Knee..

[13] Spicer DD, Blagg SE, Unwin AJ, Allum RL (2000). Anterior knee symptoms after four-strand hamstring tendon anterior cruciate
ligament reconstruction.. Knee Surg Sports Traumatol Arthrosc..

[14] Kartus J, Movin T, Karlsson J (2001). Donor-site morbidity and anterior knee problems after anterior cruciate
ligament reconstruction using autografts.. Arthroscopy..

[15] Mistry D, O'Meeghan C (2005). Fate of the infrapatellar branch of the saphenous nerve post total knee
arthroplasty.. ANZ J Surg..

[16] Mochizuki T, Muneta T, Yagishita K, Shinomiya K, Sekiya I (2004). Skin sensory change after arthroscopically-assisted anterior cruciate
ligament reconstruction using medial hamstring tendons with a vertical
incision.. Knee Surg Sports Traumatol Arthrosc..

[17] Pagnani MJ, Warner JJ, O'Brien SJ, Warren RF (1993). Anatomic considerations in harvesting the semitendinosus and gracilis
tendons and a technique of harvest.. Am J Sports Med..

[18] Poehling GaryG, Pollock FEdward, Koman LAndrew (1988). Reflex sympathetic dystrophy of the knee after sensory nerve
injury.. Arthroscopy..

[19] Bademkiran F, Obay B, Aydogdu I, Ertekin C (2007). Sensory conduction study of the infrapatellar branch of the saphenous
nerve.. Muscle Nerve..

[20] Kartus J, Ejerhed L, Eriksson BI, Karlsson J (1999). The localization of the infrapatellar nerves in the anterior knee region
with special emphasis on central third patellar tendon harvest: a dissection study on
cadaver and amputated specimens.. Arthroscopy..

[21] Aglietti P, Giron F, Buzzi R, Biddau F, Sasso F (2004). Anterior cruciate ligament reconstruction: bone-patellar tendon-bone
compared with double semitendinosus and gracilis tendon grafts. A prospective,
randomized clinical trial.. J Bone Joint Surg Am..

[22] Ejerhed L, Kartus J, Sernert N, Kohler K, Karlsson J (2003). Patellar tendon or semitendinosus tendon autografts for anterior cruciate
ligament reconstruction? A prospective randomized study with a two-year
follow-up.. Am J Sports Med..

[23] Kartus J, Ejerhed L, Sernert N, Brandsson S, Karlsson J (2000). Comparison of traditional and subcutaneous patellar tendon harvest. A
prospective study of donor site-related problems after anterior cruciate ligament
reconstruction using different graft harvesting techniques.. Am J Sports Med..

[24] Tillett E, Madsen R, Rogers R, Nyland J (2004). Localization of the semitendinosus-gracilis tendon bifurcation point
relative to the tibial tuberosity: an aid to hamstring tendon harvest.. Arthroscopy..

[25] Ebraheim NA, Mekhail AO (1997). The infrapatellar branch of the saphenous nerve: an anatomic
study.. J Orthop Trauma..

